# Severe colchicine poisoning treated successfully with kidney replacement therapy and plasmapheresis: a case report

**DOI:** 10.1080/24734306.2022.2055817

**Published:** 2022-03-24

**Authors:** D. H. Schaffer, D. L. Overbeek, T. B. Erickson, E. W. Boyer, C. Goldfine, S. A. Muhsin, P. R. Chai

**Affiliations:** a Department of Emergency Medicine, Brigham and Women’s Hospital, Boston, MA, USA;; b Department of Emergency Medicine, University of Rochester Medical Center, Rochester, NY, USA;; c Harvard Humanitarian Initiative, Cambridge, MA, USA;; d Department of Emergency Medicine, The Ohio State University Wexner Medical Center, Columbus, OH, USA;; e Renal Division, Department of Medicine, Brigham and Women’s Hospital, Harvard Medical School, Boston, MA, USA;; f Department of Psychosocial Oncology and Palliative Care, Dana Farber Cancer Institute, Boston, MA, USA;; g The Koch Institute for Integrated Cancer Research, Massachusetts Institute of Technology, Cambridge, MA, USA;; h The Fenway Institute, Boston, MA, USA

**Keywords:** Colchicine, overdose, plasmapharesis, case report

## Abstract

Colchicine is commonly prescribed for treatment of inflammatory conditions but has a narrow therapeutic window and dangerous toxicity profile. Here we describe a case of survival after massive unintentional colchicine overdose treated with plasmapheresis and renal replacement therapy. A 37 year old male with history of pericarditis presented to the Emergency Department with a chief complaint of nausea, vomiting, and diarrhea after unintentionally ingesting 36 mg of colchicine 17 h prior to arrival. An initial colchicine concentration resulted at 5.1 ng/mL (30 h post-ingestion) and peaked at 12 ng/mL (40 h post-ingestion). He was treated with continuous kidney replacement therapy (CKRT) beginning on his first day of hospitalization and with plasmapheresis on hospital days two through four. The patient’s course was complicated by multiorgan failure including coagulopathy, respiratory failure, neuropathy, renal failure, pancytopenia, and heart failure. He was discharged to inpatient rehabilitation on hospital day 24. On clinical follow up four months after discharge the patient was found to have no significant persistent morbidity related to colchicine overdose.

## Introduction

Colchicine, derived from the autumn crocus (*Colchicum autumnale*), is a common first-line agent in the treatment of inflammatory conditions such as pericarditis and gout. Colchicine has a narrow therapeutic window with potential for significant adverse events. Toxicity may occur both in therapeutic dosing as well as overdose. We describe, in accordance with the CARE Guidelines (www.care-statement.org), severe colchicine poisoning treated with kidney replacement therapy and plasmapheresis, with survival to hospital discharge.

## Case report

A 37 year-old male with a history of chronic idiopathic pericarditis and subtotal pericardial resection five years prior presented from home to a tertiary care Emergency Department (ED) with a chief complaint of nausea, vomiting, and diarrhea. The patient reported that he unintentionally ingested 60 tablets of 0.6 mg colchicine 17 h prior to arrival. The patient indicated that he keeps his medications in a single pill bottle, and accidentally took his full supply of colchicine rather than his evening medications. He denied suicidal ideation. He drank large quantities of water in an attempt to eliminate drug but developed progressive nausea and vomiting. Over the next twelve hours he experienced multiple episodes of non-bloody diarrhea and subsequent abdominal pain resulting in his ED presentation.

On arrival, vital signs were a heart rate of 101 beats per minute, blood pressure of 125/62 mmHg, respiratory rate of 16 breaths per minute, oxygen saturation of 98% on room air, and an oral temperature of 36.4 °C. On exam, the patient was well-appearing and in no distress. His eyes were anicteric without nystagmus. Neurologic exam demonstrated that the patient was alert and oriented, with intact cranial nerves, normal strength and sensation throughout all four extremities, and without evidence of ataxia. He had normal heart sounds and lungs were clear to auscultation bilaterally. His abdomen was soft but with mild diffuse tenderness most prominent in the right and left lower quadrants. There was no rebound or guarding. His skin was warm with no rash.

**Figure 1.**
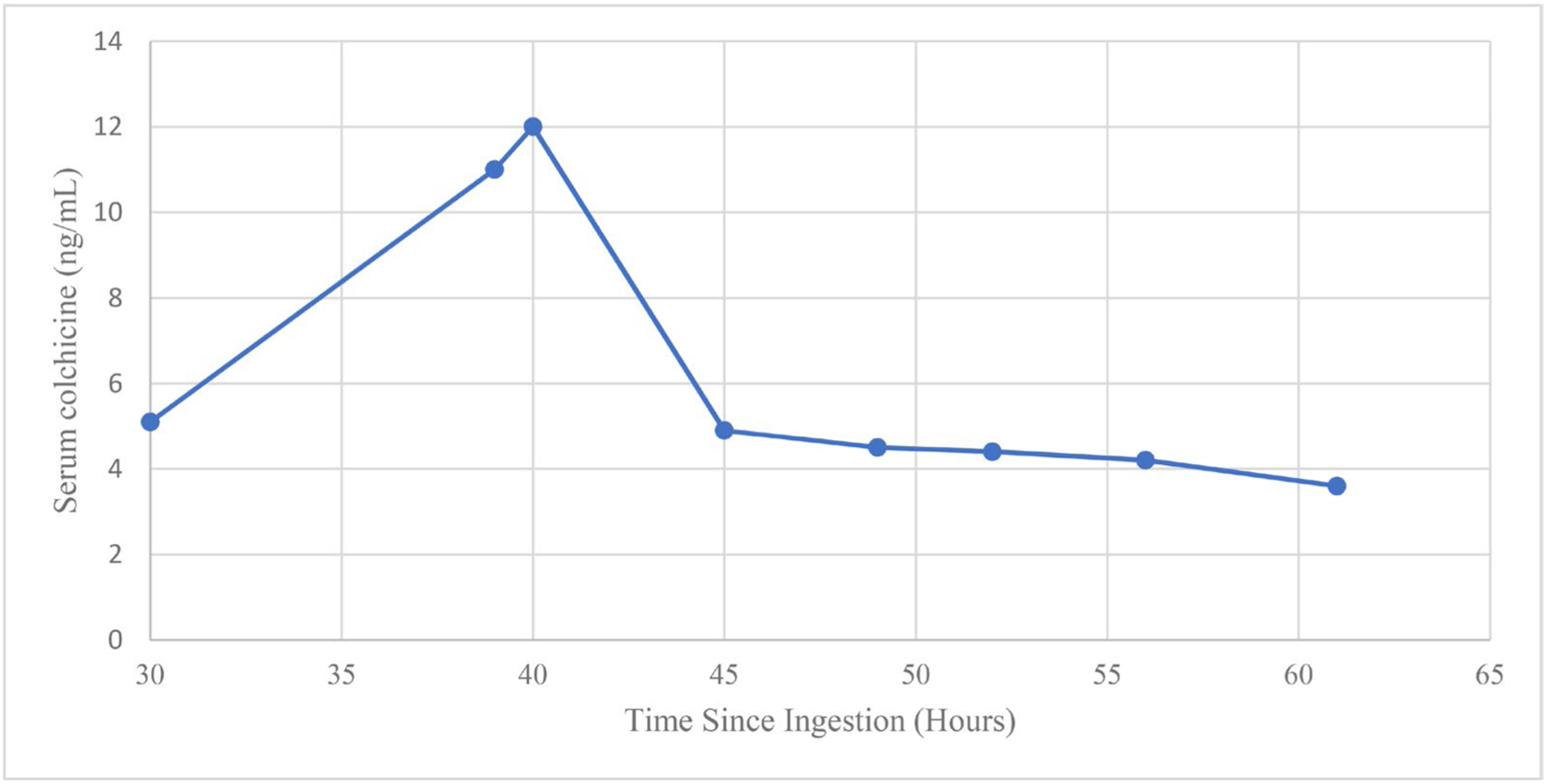
Available colchicine concentrations. The patient presented at approximately 17 h after ingestion and serum colchicine concentrations were first obtained at hour 30. Continuous kidney replacement therapy was performed for 7 days beginning at hour 27. Three cycles of plasmapheresis were performed beginning at hour 51.

An electrocardiogram (ECG) demonstrated normal sinus rhythm with PR, QRS, and QTc intervals of 160 ms, 88 ms and 436 ms, respectively. A portable plain radiograph of the chest demonstrated no acute abnormalities. Initial laboratory analysis was remarkable for a white blood cell count of 32.98 K/μl, creatinine of 2.11 mg/dL, (increased from prior of 1.12 mg/dL), serum bicarbonate of 12 mmol/L, anion gap of 28 mmol/L, aspartate transaminase (AST) of 150 U/L, alanine transaminase (ALT) of 42 U/L, lactic acid of 7.1 mmol/L, and venous pH of 7.21 (Table 1). Serum colchicine concentrations, drawn at several intervals post-ingestion, demonstrated an initial colchicine concentration of 5.1 ng/mL at 30 h post-ingestion, and a peak colchicine concentration of 12 ng/mL at 40 h post-ingestion (Figure 1).

In the ED, the patient developed frequent episodes of diarrhea with dark red blood. We elected not to perform gastrointestinal decontamination due to elapsed time since ingestion and persistent nausea and vomiting. We administered N-acetylcysteine in the Emergency Department given concern for possible hepatotoxicity. He was admitted to the intensive care unit (ICU) for continued management of his toxicity.

In the ICU, the patient underwent continuous kidney replacement therapy due to persistent acidosis, which began on hospital day 1 and was ultimately required until hospital day 8. Transthoracic echocardiography (TTE) was performed demonstrating an estimated left ventricular ejection fraction of 30% with diffuse hypokinesis, decreased right ventricular systolic function with tricuspid annular plane systolic excursion (TAPSE) of 1.2 cm. These findings of cardiac dysfunction were new from prior TTE approximately 5 years prior.

On hospital day two the patient reported new distal paresthesias and cyanopsia. He then developed increasing respiratory distress, somnolence, and was intubated. His platelet count and fibrinogen concentrations began to decline, his INR increased, and his D-dimer began to rise (Table 1). He then underwent plasmapheresis for potential improvement in colchicine clearance, and received a total of three rounds of plasma exchange between hospital days 2 through 4. He was also found to have profound lymphopenia with an absolute lymphocyte count of 0.00 K/μL. He received multiple transfusions of platelets, fresh frozen plasma, red blood cells, and cryoprecipitate between hospital days 2 and 6, at which time his coagulopathy resolved. Norepinephrine, epinephrine, and vasopressin infusions were required on hospital days 2 through 5 due to mixed cardiogenic and distributive shock.

On hospital day three a repeat TTE demonstrated new moderate to severe tricuspid valve regurgitation and moderate mitral valve regurgitation. His high-sensitivity troponin peaked at 2055 ng/L (reference range: 0 − 14 ng/L). He also developed rhabdomyolysis with a peak creatine kinase of 5151 U/L (reference range: 39 − 308 U/L), with elevated serum creatine kinase levels for the duration of his ICU course.

After extubation on hospital day 7, the patient developed fever with radiographic findings of pneumonia for which he received IV cefazolin without requiring further respiratory support. On hospital day 11 the patient developed new atrial flutter, followed by atrial fibrillation with rapid ventricular response for which he received amiodarone infusion. He was transferred out of the ICU on hospital day 14. Over the following 10 days the patient developed ileus but was discharged to inpatient rehabilitation on hospital day 24. At four months after hospital discharge, repeat TTE demonstrated improvement in biventricular systolic function. Clinic visits indicate the patient has had resolution of symptoms and no significant ongoing morbidity.

## Discussion

This case demonstrates remarkable survival of an individual with unintentional poisoning from colchicine. Prudent administration of supportive care through vasopressors, renal replacement therapy, transfusion of blood products and management of sepsis resulted in survival with minimal sequelae. This demonstrates that with efficient supportive care, survival after significant colchicine overdose is feasible.

Colchicine, a lipid-soluble alkaloid derived from *Colchicum autumnalae* (Autumn Crocus), prevents microtubule assembly by direct binding to tubulin protein. Secondary mechanisms of action include inhibition of neutrophil chemotaxis and adhesion, and possibly prevention of neutrophil superoxide production [1]. These effects result in the reduction of inflammation that makes colchicine effective in the treatment of common inflammatory conditions such as acute gout and, more recently, non-bacterial pericarditis. Arrest of mitosis caused by colchicine results in the classically described phases of acute colchicine overdose [2,3]: phase 1 (0–24 h) includes nausea, vomiting and diarrhea, as well as peripheral leukocytosis; phase 2 (1–7 days) includes pancytopenia and/or DIC and multi-organ failure, with risk of ARDS, rhabdomyolysis, renal failure, and cardiac arrhythmias or sudden cardiac death. Sepsis is common in individuals given the significant lymphopenia that can occur with toxicity. In survivors, this is followed by phase 3 (> 7 days), at which point alopecia and myopathies or neuropathies may occur.

In a previous case series, the dose of ingested colchicine was found to be predictive of outcomes: no fatalities were observed after ingestion of less than 0.5 mg/kg, 10% of cases resulted in fatality at doses of 0.5 − 0.8 mg/kg, and 100% fatality occurred at doses greater than 0.8 mg/kg [4,5]. This prediction rule, however, may not be reliable, as other cases have been described which violate these expected outcomes [6–8]. In this case, the patient progressed through the triphasic syndrome, including early gastrointestinal manifestations and peripheral leukocytosis, DIC, rhabdomyolysis, sepsis, and arrhythmia, after ingestion of 0.6 mg/kg of colchicine.

Management of colchicine overdose begins with supportive care; however, some adjunctive therapies have been proposed. Patients who present early after ingestion may benefit from GI decontamination, including activated charcoal or gastric lavage, and multidose activated charcoal may be useful even at later stages given some evidence of enterohepatic recirculation [9]. In this case, activated charcoal and lavage were avoided due to delay of presentation after ingestion and persistent vomiting.

Kidney replacement therapy for removal of colchicine appears unlikely to result in significant benefit due to the xenobiotic’s large volume of distribution, but there is some limited evidence that CKRT results in some removal of colchicine [10]. Our patient’s serum colchicine concentration dropped precipitously after initiation of CKRT (Figure 1), which may indicate removal from the serum; however, further data is needed to support this finding. As in this case, kidney replacement therapy is often necessary due to renal dysfunction, and should commence early in severe cases to maximize any benefit from improved xenobiotic clearance. Plasma exchange is also of unproven benefit in colchicine poisoning. Colchicine is an estimated 40–50% plasma protein-bound at therapeutic doses, providing theoretical support for possible benefit of plasma exchange, and two cases suggest benefit of plasma exchange in acute colchicine poisoning [8,11]. Our patient received three plasmapheresis treatments starting on day 2.

After an initial peripheral leukocytosis, bone marrow suppression resulting in neutropenia or pancytopenia is a common complication of colchicine poisoning. When neutropenia occurs, granulocyte colony-stimulating factor (G-CSF) may shorten the duration of neutropenia and may reduce the likelihood of sepsis [9,12,13]. While our patient developed coagulopathy and lymphopenia, he did not develop neutropenia and G-CSF was not administered.

Cardiovascular toxicity in colchicine poisoning can result in dysrhythmia, hypotension, and cardiac arrest [7,9]. Extracorporeal life support has been used in cases of severe colchicine poisoning associated with cardiovascular collapse, though in one case series, no patients survived [14]. Our patient developed new atrial fibrillation and atrial flutter, as well as mixed distributive and cardiogenic shock managed with three vasopressors.

A single case report described compassionate use of experimental colchicine-specific Fab antibody fragments 40 h after ingestion. Here total serum colchicine concentration abruptly increased while her free serum colchicine concentration abruptly fell. This reflects removal of colchicine from intracellular space [15]. The Fab fragments also appeared to result in significant hemodynamic improvement. An animal model also demonstrated significant improvement in removal of free plasma colchicine and mortality [16,17]. Anti-colchicine fab fragments, however, remain experimental at this time.

## Conclusion

Colchicine overdose represents an uncommon but life-threatening syndrome characterized by progressive multi-organ dysfunction, including bone marrow aplasia, cardiac toxicity, rhabdomyolysis, sepsis, and ARDS. Aggressive supportive care including early initiation of continuous kidney replacement therapy and plasmaphresis may be necessary to manage the expected course of toxicity due to colchicine poisoning.

## Figures and Tables

**Table 1.** Selected laboratory values of patient.

	Reference Values	Time since ingestion (Hours), Time and Date							
		18 h*	27 h	38 h	50 h	57 h	78 h	124 h	169 h
Laboratory Values									
Sodium (mmol/L)	136 – 145	140	137	135	133	133	135	135	139
Carbon Dioxide (mmol/L)	22 – 31	24	17	19	18	13	21	22	23
Anion Gap	7 – 17	14	16	13	13	22	13	10	12
BUN (mg/dL)	6 – 23	12	18	14	11	12	10	14	27
Creatinine (mg/dL)	0.50 – 1.20	1.12	1.51	1.35	1.17	1.47	1.06	1.09	0.81
AST (SGOT) (U/L)	10 – 50	150	234	251	282	138	370	258	183
ALT (SGPT) (U/L)	15 – 41	42	78	72	64	35	144	209	166
CK (U/L)	39 – 308	716	–	2278	4624	2508	3756	1398	2384
High-sensitivity troponin T (ng/L)	0 – 14	16	–	–	–	2055	–	–	–
Lactic Acid (mmol/L)	0.2 – 2.0	7.1	3.2	4.8	3.3	7.7	4.2	1.5	0.9
WBC (K/μL)	4.00 – 10.00	32.98	19.22	14.86	13.18	10.37	7.00	4.46	10.34
Hgb (g/dL)	13.5 – 18.0	18.3	14.7	11.9	10.3	9.8	8.8	7.6	7.3
PLT(K/μL)	150 – 450	264	103	57	28	53	50	98	42
Absolute Neutrophil Count (K/μL)	1.92 – 7.6	26.71	13.84	12.63	10.02	9.23	6.23	2.72	6.41
Absolute Lymphocyte Count (K/μL)	0.72 – 4.10	2.97	1.73	0.30	0.53	0.00	0.35	0.40	1.03
PT-INR	0.9 – 1.1	1.5	3.3	4.1	2.4	1.5	1.3	1.2	1.3
Fibrinogen (mg/dL)	200 – 450	–	–	137	290	210	325	256	302
D-dimer (ng/mL)	< 500	–	–	>4000	>4000	>4000	>4000	–	>4000
EKG Parameters		11/7 12:40		11/8 02:20	11/8 17:51		11/9 17:37	11/11 03:10	11/13 03:59
QRS (ms)	< 120	88	–	88	114	–	108	98	110
QTc (ms)	< 430	436	–	452	459	–	424	520	444

ALT, alanine aminotransferase; AST, aspartate transaminase; BUN, blood urea nitrogen; CK, creatine kinase; Hgb, hemoglobin; INR, international normalized ratio; PLT, platelets; PT, prothrombin time; QTc, corrected QT interval; WBC, white blood cells;

* Ingestion occurred at 2PM on the day prior to presentation to the emergency department. The patient presented to the emergency department at 7AM, approximately 17 h since ingestion.

## References

[1] Leung YY, Yao Hui LL, Kraus VB. Colchicine-update on mechanisms of action and therapeutic uses. Semin Arthritis Rheum. 2015;45(3):341–350. 26228647 10.1016/j.semarthrit.2015.06.013 PMC4656054

[2] Santos CD, Schier JG. Colchicine, podophyllin, and the vinca alkaloids. In: Hoffman RS, Goldfrank LR, Smith SW, Lewin NA, Howland MA, Nelson LS, editors. Goldfrank’s toxicologic emergencies, Eleventh Edition, Eleventh. New York: The McGraw-Hill Companies; 2019, pp. 501–510.

[3] Milne ST, Meek PD. Fatal colchicine overdose: report of a case and review of the literature. Am J Emerg Med. 1998;16(6):603–608. 9786547 10.1016/s0735-6757(98)90228-5

[4] Bismuth C, Baud F, Dally S. Standardized prognosis evaluation in acute toxicology its benefit in colchicine, paraquat and digitalis poisonings. J Toxicol Clin Exp. 1986;6(1):33–38. 3783481

[5] Bismuth C, Gaultier M, Conso F. Medullary aplasia after acute colchicine poisoning. 20 cases. Nouv Presse Med. 1977;6(19):1625–1629. 405656

[6] Iosfina I, Lan J, Chin C, Massive colchicine overdose with recovery. Case Rep Nephrol Urol. 2012;2(1):20–24. 23197951 10.1159/000338269 PMC3482071

[7] Mullins ME, Carrico EA, Horowitz BZ. Fatal cardiovascular collapse following acute colchicine ingestion. J Toxicol Clin Toxicol. 2000;38(1):51–54. 10696925 10.1081/clt-100100916

[8] Ozdemir R, Bayrakci B, Teksam O. Fatal poisoning in children: Acute colchicine intoxication and new treatment approaches. Clin Toxicol. 2011;49(8):739–743. 10.3109/15563650.2011.610146 21910646

[9] Finkelstein Y, Aks SE, Hutson JR, Colchicine poisoning: the dark side of an ancient drug. Clin Toxicol. 2010;48(5):407–414. 10.3109/15563650.2010.495348 20586571

[10] Rahman O, Jacobi J, Peter H, Mowry J, Sohail M. Demonstration of Colchicine Clearance by Continuous Venovenous Hemofilration (CVVH) in Severe Toxicity. Am J Respir Crit Care Med. 2018;197:A6917.

[11] Demirkol D, Karacabey BN, Aygun F. Plasma exchange treatment in a case of colchicine intoxication. Ther Apher Dial. 2015;19(1):95–97. 25257122 10.1111/1744-9987.12226

[12] Harris R, Marx G, Gillett M, Kark A, Arunanthy S. Colchicine-induced bone marrow suppression: Treatment with granulocyte colony-stimulating factor. The Journal of Emergency Medicine 2000;18:435–440. 10802421 10.1016/s0736-4679(00)00160-8

[13] Lee S-H, Park S-W, Han S-K, Acute colchicine poisoning treated with granulocyte colony stimulating factor and transfusion. Korean J Crit Care Med. 2015;30(3):207–211.

[14] Baud F, Deye N, Malissin I, Inefficacy of extra-corporeal life support in four cases of acute colchicine poisoning: a preliminary report. Clin Toxicol. 2014;52:409–410.

[15] Baud FJ, Sabouraud A, Vicaut E, Brief report: treatment of severe colchicine overdose with colchicine-specific Fab fragments. N Engl J Med. 1995;332(10):642–645. 7845428 10.1056/NEJM199503093321004

[16] Fabresse N, Allard J, Sardaby M, LC–MS/MS quantification of free and fab-bound colchicine in plasma, urine and organs following colchicine administration and colchicine-specific fab fragments treatment in göttingen minipigs. J Chromatogr B Anal Technol Biomed Life Sci. 2017;1060:400–406. 10.1016/j.jchromb.2017.06.034 28667924

[17] Eddleston M, Fabresse N, Thompson A, Anti-colchicine fab fragments prevent lethal colchicine toxicity in a porcine model: a pharmacokinetic and clinical study. Clin Toxicol (Phila). 2018;56(8):773–781. 29334816 10.1080/15563650.2017.1422510 PMC6021765

